# Prevalence and patterns of traditional bullying victimization and cyber-teasing among college population in Spain

**DOI:** 10.1186/s12889-016-2857-8

**Published:** 2016-02-19

**Authors:** Francisco Caravaca Sánchez, María Falcón Romero, Javier Navarro-Zaragoza, Aurelio Luna Ruiz-Cabello, Oriali Rodriges Frantzisko, Aurelio Luna Maldonado

**Affiliations:** Department of Social and Health Sciences, University of Murcia, 30100 Campus of Espinardo, Murcia, Spain

## Abstract

**Background:**

Traditional bullying victimization and the growing number of cyber-teasing victims during the last decade is a major public health concern. The objective of this study was to examine the relationship between students’ experiences of traditional bullying victimization and cyber-teasing and the sociodemographic characteristics of a sample composed of college students in Spain.

**Methods:**

In the fall of 2014, 543 sixth-grade students from southeast Spain completed an anonymous survey on their experience of both kinds of to ascertain any relationship with sociodemographic characteristics, including gender, nationality, economic problems, family conflicts and alcohol and cannabis use.

**Results:**

A total of 62.2 % of the students reported to having suffered traditional bullying victimization and 52.7 % reported that they had been subject to cyber-teasing. 40.7 % of participants had been victims of traditional bullying victimization and cyber-teasing in the past 12 months. Most (65.7 %) of the victims were at the same time cyber-teasing victims; 77.6 % of cyber-teasing victims were also victimized in a different manner. Traditional bullying victimization was higher among boys than among girls, while female students were more likely to have been subjected to cyber-teasing than male students. The characteristics that most heavily influenced suffering traditional bullying victimization were economic problems, family conflicts and cannabis use.

**Conclusions:**

Our findings confirm overlapping results in the risk factors that influence suffering both traditional bullying victimization and cyber-teasing: there was a strong influence of certain sociodemographic and individual characteristics of the college population, suggesting that specific policies are necessary to improve college students’ environment in Spain.

## Background

In the recent years, awareness of new young victims of cyber-bullying has increased [1, 2] due to the *boom* in information and communications technology and the use of social network. The internet and other electronic media offer many benefits to adolescents, even though previous cross-sectional studies have shown that cyber-bullying and cyber-teasing among the school population is associated with mental health problems such as depression, suicidal thoughts and non-fatal suicidal behavior among school students [3–5].

Cyber-bullying, also known as electronic bullying or online bullying is defined as intentional and repeated harm inflicted on individuals through the use of electronic devices such as mobile phones or computers [6]. Similar to definitions of traditional bullying, cyber-bullying also implies that the offender’s behavior is usually aggressive with intention to harm the victims [7]. Cyber-teasing is a related phenomenon defined as hurtful teasing sent via technology that includes messages resulting in psychological abuse, verbal aggression or social rejection that has received less attention that cyber-bullying [8].

Previous studies have found rates of cyber-bullying among young regular Internet users (between 10 and 17 years old) that range from 4 to 72 % [9, 10], and lower rates among college population compared to adolescent samples ranging from 9 to 20 % [11–14]. Cyber-bullying and cyber-teasing are known to cause higher level of depression disorders in victims that traditional ways of bullying among adolescents [15]. Finally, victims frequently suffer additional problems involving aggressiveness, substance use or delinquency [15].

Cyber-bullying can occur in several ways [16], and its effects are intensified by the audience reached or the ability to attack at any time or in any place [7, 15]. Nevertheless, schools still lack of information on the effects and consequences of this problem, which they tend not to distinguish from traditional bullying [7].

According to Olweus [17], bullying is defined as a repeated negative behavior that occurs along time in a relationship characterized by an imbalance of strength and power. However, approximately three decades of studies on bullying victimization have led to different definitions of bullying [18]. Bullying and victimization are two of the most significant health problems among adolescents, with an international prevalence that ranges from 9 to 54 % [19, 20] and from 10 to 25 % among college population [21, 22]. Thus, traditional bullying victimization does not disappear in college; although it is less prevalent than among younger students [15, 19]. A cross-sectional study conducted in 40 countries showed that approximately 25 % of adolescents suffer bullying or victimization with some regularity [23]. Earlier studies also found a high prevalence of traditional bullying victimization among high school and college population [24–26].

An analysis of the relationship between traditional bullying victimization and cyber bullying, conducted recently by Schneider and colleagues [25] among high school students found an overlap between both types of victimization (traditional bullying victimization and cyber bullying), whereby 59.7 % of cyber-bullying victims were also school traditional bullying victims, and 36.3 % of school traditional bullying victims were also cyber-bullying victims. Further information about this relation is difficult to find in the literature, especially in the case of college population. Previous studies [9, 27, 28] showed that between 1/3 and 3/4 of school and high school students bullied online were also victims of traditional bullying.

Previous research also documented the high prevalence of drug use among college students [29] especially in the case of marijuana [30, 31] and alcohol [32, 33]. In 2011 in the United States 64 % of college students reported they had drunk alcohol in the month previous to the survey [29]. Different studies among youths [34, 35] have examined the association between substance use and traditional bullying victimization and cyber bullying, finding that bully-victims have a higher risk of drinking alcohol and to substance use compared with their equals that do not suffer any kind of traditional bullying victimization. Finally, in the family context, conflict at home and low socioeconomic status have been traditionally established as risk factors of violence and traditional bullying victimization in adolescents [36 - 39].

### Research questions

Recent studies have shown a high prevalence of traditional bullying victimization and cyber-teasing among adolescents and the college population worldwide, including America [16, 32], Asia [2, 40] and Europe [41–45]. Nevertheless, the literature that analyzes the associations between traditional bullying victimization and cyber-teasing is limited. Based on the previous literature [15, 16, 19, 21] we hypothesized that prevalence of traditional bullying victimization and cyber-bullying will be lower among our college population sample. The purpose of the current study is to study the prevalence of traditional bullying victimization and cyber-teasing and their association among college students in the southeast of Spain, and to analyze the influence of individual and sociodemographic characteristics on the risk of suffering traditional bullying victimization and cyber-teasing among college population.

## Method

### Participants

The participants were 543 college students studying from 2^nd^ to 6^th^ years in the University of Murcia, with an average age of 22.6 years (SD = 6.01). The distribution of the sample reflected the distribution of the total population studying at the University of Murcia (*n* = 34,404) during the 2013–2014 course. In our case, 8.1 % were overseas students (with a mean age of 23.7), which is similar to the proportion of foreign students at the University (9.7 %). Approximately a quarter (25.4 %) of the sample was male (with a mean age of 23.5) while the proportion for male students at the University of Murcia as a whole is 32 %.

This bias for gender might be explained in part by the higher proportion of female college students in Spain assuming ≈ 65 % according to the “Instituto Nacional de Estadistica” [46]. But also because the higher proportion of female students in the degree of Medicine in Spain (72 % and approximately 70 % at the University of Murcia [46].

### Data collection

A stratified random sample was selected from the university. Participants were medical students from the 2nd grade to 6th years in two courses of the University of Murcia (*n* = 607), who volunteered to participate in a self-report questionnaire collected from September and December 2014. Of those invited to participate 30 women and 44 men refused for the following reasons: “there is nothing to be gained from the survey” and “I am leaving the University soon”. Questions about traditional bullying victimization and cyber-teasing referred to the previous twelve months. For this reason, the students who were in their first year of university were excluded from the research.

College students and teachers were notified in advance via email including information about the day the survey would take place and they were given the opportunity to pre-view the survey, those who did not want to participate were excused from going to the lesson that day. An interviewer (from the Research staff of University of Murcia) remained in the classroom while the students answered the questionnaire. If participants did not understand a specific question, the interviewer re-read the question in order to make it more clear without leading them in any particular direction. Participants were not paid for taking part in the current research. The survey was voluntary and anonymous.

The surveys were administered in a classroom by 3 interviewers who received approximately 5 h of training on the data collection protocol and questionnaire by the University of Murcia. The interviewers followed a scripted protocol to explain the questionnaire and the study. Surveys were self-administered to groups of approximately 20 people who used paper and pencil to record their responses. The survey questions were written in Spanish and each group session lasted approximately half an hour. The study was approved by the University of Murcia Research Ethics Board. Signed consent forms from participants were obtained prior to the questionnaires being administered.

### Measures

Variables for the present study were selected according to the previous literature about traditional bullying victimization and cyber-bullying among different ages, in order to detect protective or risk factors linked to both types of victimization such as: foreigner [43], low socioeconomic status [38], family conflicts [37], alcohol or cannabis use [34, 35], and academic grade [25].

#### Sociodemographics

Demographic variables included age, gender (coded as 1 for girls and 0 for boys), nationality (coded as 1 for Spanish and 0 for foreigners), and grade. The respective family economic situation consisted of one item: “Currently, does your family have economic difficulties?”. Responses included “Yes” (coded as 1) or “No” (coded as 0). Questions regarding family conflicts were drawn from the Communities that Care survey which has been found to have acceptable psychometric properties [35]: “Do any people in your family often insult or yell at each other?”. Responses included “Yes” (coded as 1) or “No” (coded as 0). At the end of demographic characteristics section, questions from previous research [47] were adapted to measure alcohol and cannabis use by the following questions: “Have you consumed alcohol during the last year?” and “Have you consumed cannabis during the last year?”. Responses included “Yes” (coded as 1) or “No” (coded as 0).

#### Traditional bullying victimization and cyber-teasing

Students were asked about traditional bullying victimization and cyber-teasing in the past 12 months. Cyber-teasing was measured with the following specific question: “How many times has someone used the Internet, a phone, or any other electronic devices to bully, tease, or threaten you in the past 12 months?”. The questions regarding cyber-teasing were adapted from previous studies [25, 27]. The question regarding traditional bullying victimization was adapted from the “Olweus Bullying Questionnaire” [48, 49], specifically the question was: “How often have you been bullied in the past 12 months?”. Both questions used a 4-point response scale: “I haven’t been cyber-teased in the past 12 months” (coded as 0), “It has only happened once” (coded as 1), “It has happened twice” (coded as 2), and “It has happened more than 2 times” (coded as 3). Respondents who selected affirmative answers (coded as 1, 2 and 3) were classified as subjects that had experienced cyber-teasing or traditional bullying victimization.

### Data analysis

Data were analyzed using SPSS Statistics 20 for Windows (SPSS, IBM Inc.) adopting a 95 % (p ≤ 0.05) significance level. The study was conducted in four steps. First, univariate analysis of variance and Chi^2^ were computed to contrast the distribution by gender and demographic characteristics. Secondly, the prevalence of traditional bullying victimization and/or cyber-teasing among the participants for the whole sample and by gender, as well as Chi^2^ statistics were calculated in the search for associations by gender and traditional bullying victimization or cyber-teasing. Thirdly, cross-tabulations were used to make a bivariate analysis of both types of victimization and sociodemographic characteristics. Finally, a logistic regression analysis was developed to examine the relationship between traditional bullying victimization, cyber-teasing or both in the previous twelve months (dependent variable) and sociodemographic variables (independent variables), including gender, nationality, grade, economic problems, family conflicts, alcohol use and cannabis use in the last twelve months.

## Results

### Prevalence and overlap of traditional bullying victimization and cyber-teasing

Table 1 describes the sociodemographic characteristics of the survey college population for full sample and by gender. The prevalence of cyber-teasing and traditional bullying victimization for the whole sample and according to gender is shown in Table 2. Overall, 52.7 % (95 % confidence interval [CI]: 48.3–57.1 %) of students reported cyber-teasing, 62.2 % (95 % CI: 58.0–66.1 %) reported traditional bullying victimization during the previous twelve months, and 40.7 % (95 % CI: 36.8–44.6 %) were victims of both types of victimization (traditional bullying victimization and cyber-teasing) in the last twelve months. Analyzing the overlap between both types of victimization, there was a statistically significant difference: 65.7 % of traditional bullying victims were also victims of cyber-teasing, and 77.6 % of cyber-teasing victims suffered also traditional bullying victimization (*χ*^2^ = 60.789, *p* <0.001). Categorizing these reports into two, we obtained the following results: 21.3 % suffered traditional bullying victimization only and 12 % victims of cyber-teased only.

**Table 1:** Sociodemographics characteristics of study sample for full sample and by gender

Variables	Total	Male	Female
	*N* = 543	*N* = 138	*N* = 405
	No. (%)	No. (%)	No. (%)
Nationality			
Spanish	499 (91.9)	132 (95.7)	367 (90.6)
Non-Spanish	44 (8.1)	6 (4.3)	38 (9.4)
Grade			
2^nd^	195 (35.9)	36 (26.1)	159 (39.3)
3^rd^	144 (26.5)	26 (18.8)	118 (29.1)
4^th^	82 (15.1)	36 (26.1)	46 (11.4)
5^th^	58 (10.7)	12 (8.7)	46 (11.4)
6^th^	64 (11.8)	28 (20.3)	36 (8.9)
Economic problems***			
Yes	183 (33.7)	28 (20.3)	155 (38.3)
No	360 (66.3)	110 (79.7)	250 (61.7)
Family conflicts*			
Yes	99 (18.3)	19 (13.8)	85 (21.0)
No	444 (81.7)	119 (86.2)	320 (79.0)
Alcohol use			
Yes	438 (80.7)	111 (80.4)	327 (80.7)
No	105 (19.3)	27 (19.6)	78 (19.3)
Cannabis use**			
Yes	88 (16.2)	34 (24.6)	54 (13.3)
No	455 (83.8)	104 (75.4)	351 (86.7)

Statistically significant difference between male and female students; *p ≤ .05, **P ≤ .01, ***P ≤ .001

**Table 2:** Prevalence of cyber-teasing and traditional bullying victimization during the past twelve months in full sample and by gender

	Total	Male	Female
	*N* = 543	*N* = 138	*N* = 405
	No. (%) and 95 % CI^a^	No. (%) and 95 % CI^a^	No. (%) and 95 % CI^a^
Cyber-teasing			
None victims	257 (47.3) 42.9–51.7	73 (52.9) 44.4–60.8	184 (45.4) 40.4–50.4
Victims	286 (52.7) 48.3–57.1	65 (47.1) 39.2–55.6	221 (54.6) 49.6–59.6
1 time	137 (25.2) 21.6–29.1	33 (23.9) 16.5–31.9	104 (25.7) 21.7–29.9
2 time	45 (8.3) 5.9–10.7	14 (10.1) 5.6–15.0	31 (7.7) 5.2–10.2
More than 2 times	104 (19.2) 15.8–22.3	18 (13.0) 7.5–18.6	86 (21.2) 17.5–25.1
Traditional bullying victimization			
None victims	205 (37.8) 33.9–42.0	40 (29.0) 21.9–36.4	165 (40.7) 36.0–45.7
Victims*	338 (62.2) 58.0–66.1	98 (71.0) 63.6–78.1	240 (59.3) 54.3–64.0
1 time*	177 (32.6) 28.9–36.6	58 (42.0) 34.1–50.4	119 (29.4) 25.1–33.8
2 time	36 (6.6) 4.6–8.8	6 (4.3) 1.3–8.1	30 (7.4) 5.0–10.1
More than 2 times	125 (23.0) 19.5–26.5	34 (24.6) 17.7–32.1	91 (22.5) 18.4–26.5
Cyber and traditional bullying victimization			
None victims	322 (59.3) 55.4–63.2	77 (55.8) 47.3–63.7	245 (60.5) 55.9–65.4
Victims	221 (40.7) 36.8–44.6	61 (44.2) 36.3–52.7	160 (39.5) 34.6–44.1
1 time*	68 (12.5) 9.5–15.5	26 (18.8) 12.8–25.8	42 (10.4) 7.6–13.4
2 time	82 (15.1) 12.3–18.4	22 (15.9) 10.3–22.0	60 (14.8) 10.9–18.3
More than 2 times	71 (13.1) 10.1–16.0	13 (9.4) 4.6–14.6	58 (14.3) 10.7–17.8

^a^Confidence interval

Statistically significant difference between male and female students; *p ≤ .05

### Correlates of traditional bullying victimization and cyber-teasing

Table 3 displays the demographic characteristics of victimization when they are categorized into the following four groups: cyber-teasing victim only, traditional bullying victimization only, cyber-teasing and traditional bullying victimization, and neither. Reports of traditional bullying victimization were higher among boys than among girls (26.8 % vs. 19.5 %, *χ*^2^ = 6.052, *p* = 0.014) while cyber-teasing was more prevalent among girls than among boys (15.0 % vs. 2.8 %, *χ*^2^ = 10.352, *p* ≤ 0.001).

**Table 3:** Sociodemographic correlations of traditional bullying victimization and cyber-teasing of study sample

Variables	Cyber-teasing victim only	Traditional bullying victimization only	Cyber-teasing and traditional bullying victimization	Neither
	No. (%)	No. (%)	No. (%)	No. (%)
Gender				
Girl	61 (15.0)***	79 (19.5)*	160 (39.5)	105 (25.9)
Boy	4 (2.8)	37 (26.8)	61 (44.2)	36 (26.0)
Nationality				
Spanish	61 (12.2)	111 (22.2)**	200 (40.0)	127 (25.4)
Non-Spanish	4 (9.0)	5 (11.3)	21 (47.7)	14 (31.8)
Grade				
2°	29 (14.8)	40 (20.5)	85 (43.5)	195 (21.1)
3°	25 (17.3)	26 (18.0)	59 (40.9)	34 (23.6)
4°	2 (2.4)	20 (13.8)	33 (40.2)	27 (32.9)
5°	5 (8.6)	16 (27.5)	24 (41.3)	13 (7.5)
6°	4 (6.2)	14 (21.8)	20 (31.2)	26 (40.6)
Economic problems				
Yes	35 (19.1)**	28 (15.3)**	80 (43.7)	40 (21.8)
No	30 (8.3)	88 (24.4)	141 (39.1)	101 (28)
Family conflicts				
Yes	61 (21.3)***	70 (20.7)	51 (49.0)*	26 (25.0)
No	43 (16.7)	34 (32.6)	170 (38.7)	116 (26.4)
Alcohol use				
Yes	52 (11.8)	101 (23.0)*	179 (40.8)	106 (24.2)
No	13 (12.3)	15 (14.2)	42 (40.0)	35 (33.3)
Cannabis use				
Yes	10 (11.3)	24 (27.2)	30 (34.0)	24 (27.2)
No	55 (12.0)	92 (20.1)	191 (41.9)	117 (25.7)

Statistically significant difference between victims and non-victims; *p ≤ .05, **P ≤ .01, ***P ≤ .001

According to the nationality of the college students, traditional bullying victimization was almost twice more frequent in the case of Spanish students compared with foreign students (22.2 % vs. 11.3 %, *χ*^2^ = 8.052, *p* = 0.003). As regards the economic situation, cyber-teasing (19.1 % vs. 8.3 %, *χ*^2^ = 11.45, *p* ≤ 0.001) and traditional bullying victimization (24.4 % vs. 15.3 %, *χ*^2^ = 6.678, *p* = 0.009) was higher among participants with low financial resources. Family conflicts were also higher among college students who suffered traditional bullying victimization and cyber-teasing (49.0 % vs. 38.7 %, *χ*^2^ = 6.124, *p* = 0.016). Analyzing the substance use data, 80 % of the college students had drunk alcohol during the last year, and, among these students, traditional bullying victimization was more common than among non-drinkers (23.0 % vs. 14.2 %, *χ*^2^ = 5.831, *p* = 0.023).

Table 4 present the logistic regressions which regulate the relationship of the different ways of victimization (cyber-teasing victim only, traditional bullying victimization only, cyber-teasing and traditional bullying victimization, or neither of them) for the sociodemographic characteristics. There was a strong relationship between the grade of the students and suffering cyber-teasing only. For example, the college students of 2^nd^ (adjusted odds ratio [AOR] =3.86; 95 % CI: 1.19, 12.48, *p*-value = 0.004) and 3^rd^ grade (AOR = 3.94; 95 % CI: 1.20–12.94, *p*-value = 0.006) were almost 4 times more likely to report cyber-teasing than students of 6^th^ grade. Economic problems among the college students were also a risk factor for suffering cyber-teasing only (AOR = 2.46; 95 % CI: 1.29–4.71, *p*-value = 0.008). Finally, family conflicts were a risk factor for both cyber-teasing and traditional bullying victimization (AOR = 1.62; 95 % CI: 1.10–2.34, *p*-value ≤ 0.001).

**Table 4:** Associations between traditional bullying victimization and cyber-teasing and sociodemographic characteristics of study sample

Characteristics	Cyber-teasing victim only	Traditional bullying victimization only	Cyber-teasing and traditional bullying victimization	Neither
	AOR^a^ (95 % CI^b^)	AOR^a^ (95 % CI)	AOR (95 % CI)	Ref.
Gender				
Girl	0.26 (0.08–0.79)	1.49 (0.84–2.64)	1.34 (0.81–2.23)	1.00
Boy (ref)	1.00	1.00	1.00	1.00
Nationality				
Spanish	1.92 (0.59–6.24)	2.50 (0.86–7.23)	1.08 (0.52–2.23)	1.00
Foreigner (ref)	1.00	1.00	1.00	1.00
Grade				
2°	3.86 (1.19–12.48)*	2.10 (0.96–4.68)	2.92 (1.44–5.94)*	1.00
3°	3.94 (1.20–12.94)*	1.60 (0.69–3.73)	2.44 (1.17–5.05)*	1.00
4°	0.50 (0.84, 3.01)	1.46 (0.61–3.52)	1.61 (0.64–3.50)	1.00
5°	2.14 (0.48–9.51)	2.67 (0.98–7.22)	2.60 (1.05–6.43)*	1.00
6° (ref)	1.00	1.00	1.00	1.00
Economic problems				
Yes	2.46 (1.29–4.71)**	1.54 (0.70–2.51)	1.17 (0.72–1.91)	1.00
No (ref)	1.00	1.00	1.00	1.00
Family conflicts				
Yes	1.34 (0.87–2.08)	1.31 (0.83–2.06)	1.62 (1.10–2.34)*	1.00
No (ref)	1.00	1.00	1.00	1.00
Alcohol use				
Yes	0.84 (0.38–1.87)	1.63 (0.80–3.31)	1.16 (0.66–2.03)	1.00
No (ref)	1.00	1.00	1.00	1.00
Cannabis use				
Yes	1.61 (0.48–2.80)	1.31 (0.66–2.60)	0.74 (0.39–1.40)	1.00
No (ref)	1.00	1.00	1.00	1.00

^a^Adjusted Odds Ratio;^b^Confidence interval

Statistically significant difference between victims and non-victims; *p ≤ .05, **P ≤ .01

## Discussion and conclusions

The aim of the present study was to analyze for the first time in Spain the prevalence and the different characteristics of traditional bullying victimization and cyber-teasing in a university population, and to assess how sociodemographic characteristics might be a protective or risk factor for suffering both types of violence. Data from this research support and extend findings from previous studies about the nature and prevalence of traditional bullying victimization and cyber-teasing. More specifically, we found that over a half of the subjects suffered traditional bullying victimization (62.2 %), cyber-teased (52.7 %), or had suffered both cyber-teasing and traditional bullying victimization (40.7 %) during the past 12 months.

Previous studies among adolescent and college students in United States and European countries such as Finland and Sweden observed substantially lower prevalence of traditional bullying victimization or cyber-teasing than we did (e.g. cyber-bullying: approximately 7 % and 5 %) [16, 41, 43]. However, other cross-national studies in Spain among adolescents between 13 and 17 years old [45] and in other countries have found a similar prevalence of traditional bullying victimization or cyber-teasing to that which we observed, ranging from 24 to 40 % over a 6- to 12-month time period [16, 42]. Methodological differences, more specifically the definition and measure of traditional bullying victimization and cyber-teasing and the time frame of this research contributed to the variation in estimated rates [50].

Our results show that prevalence of cyber-teasing was higher than traditional bullying victimization; however, most of the previous studies reported that cyber-teasing was much less prevalent than traditional bullying victimization among school and adolescents students [3, 15, 25, 51]. It should be noted that in the current research teasing was included in the measure for cyber-bullying, but not for victimization, which might explain this finding. In fact, a previous study showed that teasing is one of the most common types of victimization among adolescents’ samples [15, 52].

We found that most cyber-teasing victims also reported that they experienced traditional bullying victimization (and *vice versa*) during the previous twelve months, suggesting that there is a clear overlap between traditional bullying victimization and suffering cyber-teasing in at any moment. In fact, 65.7 % of traditional bullying victims were also cyber-teasing victims, and 77.6 % of cyber-teasing victims also suffered traditional bullying victimization. This finding is consistent with previous literature about studies in school and high school students [27, 28].

Our results also found differences according to gender, in agreement with previous studies in adolescents [15, 53] and college [19] students, female being more likely than male students to report cyber-teasing. Perhaps this finding can be explained by the fact that female university students are more likely to use their mobile phone [54]. Indeed the increased use of mobile phones in recent years has been linked to suffering cyber victimization among adolescents [55]. Rates of traditional bullying victimization were higher among boys than girls (71 % vs. 59.3 %), while previous studies showed the opposite [56]. However high school girls are more likely to run for conflict resolution strategies [57].

An interest finding was that Spanish students were more often victimized (traditional bullying victimization only or cyber-teasing only) than foreigner students, what is contradictory to previous research from Finnish adolescents [43] or school students in the United States [58]. Perhaps this finding might be explained by the socioeconomic status: exchange college students may be more wealthy than Spanish students, but school and young foreigner students and their families might have a lower socioeconomic background. This difference between foreigner college students and foreigner school students is explained because of the phenomenon of immigration which implicates poverty. In fact, in the current research, economic position was associated with both ways of victimization, as seen in earlier research conducted in children [52] and adolescents [59]. Family conflicts were also associated with traditional bullying victimization and cyber-teasing during the last twelve months, coinciding with previous research developed in schools [36].

Finally, our data support previous research that found high rates of legal and illegal drug use among college students [29, 60]: 80.7 % of our sample population had drunk alcohol and 16.2 % smoked cannabis during the previous twelve months at university. Contrarily to previous research, alcohol or cannabis use were not a predictor of suffering traditional bullying victimization and/or cyber-teasing in this study [26, 61, 62].

### Limitations and strengths of the study

This study has a certain number of limitations. Firstly, because we were unable to use a longitudinal design, a cross-sectional study was conducted, so that it was only possible to measure the study variables at a given time and not follow their evolution. Secondly, our time period referred to the last 12 months, whereas most research refers to the past 6 months [48] and the last 2 months [15], which would explain a higher frequency of traditional bullying victimization or cyber-teasing. We decided to use a longer time frame than previous studies among adolescents because we expected a lower prevalence of traditional bullying victimization and cyber-teasing in college students based on previous studies [15, 16, 19]; nevertheless, we found a high prevalence of both types of victimization. Thus our hypothesis was not sustained. Thirdly, we did not have the possibility to analyze psychological and physical consequences of traditional bullying victimization and cyber-teasing in our sample [63]. Moreover, the demographics of our sample were limited by gender distribution (because of the low representation of males, approximately 25 %), potentially limiting the generalizability of our results. However, the prevalence of women students in the current research is similar to previous studies among college population [13]. Despite these limitations, this study addresses gaps in the literature and is one of the first to examine the association among traditional bullying victimization and cyber-teasing, and the risk factors associated to these problems among Spanish college students.

The main strength of the present study is the fact that it is the first carried out in Spain to examine traditional bullying victimization and cyber-teasing among college students, so that the results and conclusion might serve as a guide for future research in our country to examine and prevent victimization among this collective. Another important strength of the study is that was developed among a college population, whereas most studies worldwide on victimization and cyber-bullying have targeted elementary, middle, and high school students [16, 25, 64–66]. Although there is a large number of programs aimed at reducing traditional bullying victimization and cyber-bullying among youth in the United States [9, 67], Asia [2], Europe [68], and specifically Spain [69], it is still not clear what techniques and programs would be effective in order to reduce traditional bullying victimization and cyber-bullying because of the few representative studies focused on this population [16, 70]. Nowadays, different prevention programs are taking place worldwide in order to increase traditional bullying victimization and cyber-bullying knowledge such as the Olweus Bullying Prevention Program [71], the KiVa anti-bullying programme [72] or “zero tolerance” policies [73] demonstrating reductions in both types of victimization for different age groups. However, studies about the patterns and programs on college samples are underdeveloped compared to younger generations. Besides, very often these programs are not relevant because they were developed in schools [74] in spite of that previous studies have shown that cyberbullying in high school may also lead to cyberbullying during college [12, 75]. Moreover, the few specific prevention programs and awareness campaigns created in universities by different developers (counselling office, student organisations [76] or video program [70] were effective and reduced traditional bullying and cyber-bullying rates. Furthermore, prevention and intervention programs should not be limited to school and adolescents samples, and should be extended to college students.

### Practice and policy

In summary, the current research increases our knowledge of the association between traditional bullying victimization, cyber-teasing and demographic and behavioral characteristics. These findings have several implications for interventions. Regarding the prevention, there is an association of both types of victimization with other risk behaviors such as substance use and family conflicts (including economic problems). Given that, it could be important to include strategies to prevent bullying based on these patterns. Those strategies could be implemented in college age students, providing counseling services for legal and illegal substance use.

Future research with longitudinal designs is needed in order to investigate associations between traditional bullying victimization and cyber-teasing and subsequent exposure to demographics and substance risk factor exposure, as well as to examine the relationship between cyber-teasing and traditional bullying victimization (and *vice versa*) among college population, who show similar patterns and risk factors to adolescent samples [23, 35, 38]. Previous studies in this field have shown that traditional bullying victimization and cyber-teasing are an increasing phenomenon and a growing health problem among young people. This study found a strong association between traditional bullying victimization and cyber-teasing (and *vice versa*), as well as the importance of demographics and individual characteristics as risk factors associated.
